# Meeting the Shortage of Human Cells and Tissues: The Andalusian Quality Assurance Programme for Tissue Donation

**DOI:** 10.3389/ti.2024.12627

**Published:** 2024-05-01

**Authors:** Antonia José Alvarez-Marquez, Jesus Huet, José Miguel Pérez-Villares, Domingo Daga-Ruiz, Concepción Diaz-Aunión, Pablo Castro de la Nuez, Natividad Cuende

**Affiliations:** ^1^ Andalusian Transplant Coordination, Andalusian Health Service, Seville, Spain; ^2^ Servicio de Medicina Intensiva, Hospital Universitario Virgen de las Nieves, Granada, Spain; ^3^ Servicio de Medicina Intensiva, Hospital Virgen de la Victoria, Málaga, Spain

**Keywords:** critical pathway, tissue donation, tissue establishments, tissue procurement, quality assurance programme

## Abstract

**Background** A quality assurance programme for the tissue donation process was launched in Andalusia in 2020 to facilitate the integration of tissue donation into end-of-life care, and to respond to the growing need for human tissue for therapeutic purposes. The results of this programme are presented here.

**Methods** After identifying the hospital departments in which to intensify the detection of tissue donors, expanding training activities and designing a specific data collection system for possible tissue donors who do not donate their tissues, the results of the donation activity were quantified and the causes of non-donation were analysed by applying the critical pathway for deceased tissue donation methodology.

**Results** After an initial drop in activity, which coincided with the coronavirus pandemic, the number of tissue donors increased by 48.4% in 2022 compared to 2019. From the eligible donors, 83% were actual tissue donors and 71% were utilised donors. The modifiable causes of tissue donation loss, in order of frequency, were family refusal, followed by organisational or logistical issues, failure to notify or failure to identify possible donors, and failure to complete donor evaluation.

**Conclusion** As a result of the collaboration of the various professionals involved in the programme, tissue donation activity has increased remarkably, the potential and effectiveness of the donation process have been evaluated, and areas for improvement have been identified, which we hope will lead to continuous improvement of the process.

## Graphical Abstract

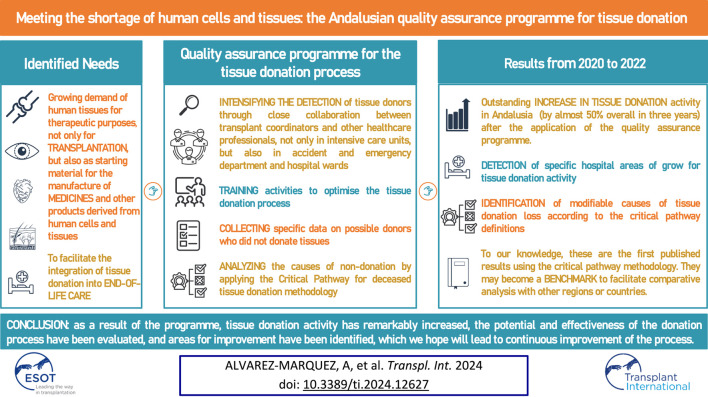

## Introduction

Spain has been the world leader in organ donation for transplantation for the last three decades [1] and Andalusia, Spain’s largest region with a population of almost 8.5 million inhabitants, achieves donation rates that are usually above the Spanish average [2]. The Spanish and Andalusian success derives from a specific organizational approach, the so-called Spanish model [3].

The key element of the Spanish model is the figure of the transplant coordinator (TC) appointed at each procurement hospital. The TCs, responsible for developing a proactive donor detection programme and effectively converting potential into actual donors, are in-house professionals and members of staff of the procurement hospital concerned. They are nominated by and report to the medical direction of the hospital, and therefore do not report to the transplantation team. Most of the TCs are involved in donation activities on a part-time basis, which enables them to be appointed even at hospitals with low deceased donor potential. Notably, a majority of TCs are critical care physicians so their daily work is carried out precisely in those units where more potential donors are detected [3].

The Spanish quality assurance programme for the organ donation process, launched in 1999 after a pilot programme in Andalusia and several other Spanish regions in 1998, has been another key factor in helping Spain maintain this position. This programme allows potential areas for improvement to be identified, with the aim of implementing measures to increase donation rates according to each hospital’s potential and characteristics [4, 5].

With respect to human tissue donation, in the last two decades Andalusia has progressively increased the donation rates, reaching the highest donation activity in 2019, although insufficient to meet the demand for human tissues. For that reason, the Regional Transplant Coordination of Andalusia designed and promoted a quality assurance programme for the tissue donation process by adapting the methodology of the quality assurance programme for organ donation. The reason for this was to achieve self-sufficiency [6, 7] in the face of the growing need for human tissue donation, not only for transplantation, but also as starting material for the manufacture of medicines and other products derived from human cells and tissues.

Facilitating tissue donation for every patient who dies in hospital, and thus truly integrating donation at the end-of-life into hospital care, is another equally important aim of this quality assurance programme for the tissue donation process [8, 9]. In order to achieve this goal, it is essential to raise awareness of this issue among healthcare professionals [10].

The programme was launched in January 2020 and is based on three principles: i) intensifying the detection activities for possible tissue donors in certain hospital departments, with a special focus on cornea donation, ii) training to optimise the tissue donation process and cornea procurement, and iii) a system, which is progressively being implemented in hospitals in Andalusia, for collecting specific data on possible donors who do not donate their tissues to identify modifiable causes of donation loss.

This manuscript illustrates how the results obtained in Andalusia regarding tissue donation have evolved from 2019, the year before the programme was implemented, to 2022. Additionally, the reasons for non-donation of tissues in the last year have been analysed using the definitions in the critical pathway for deceased tissue donation, developed by the European Committee on Organ Transplantation of the Council of Europe (CD-P-TO) published in 2021 [11], in order to obtain information that is comparable with other regions or countries that have implemented the critical pathway for deceased tissue donation methodology.

## Materials and Methods

### Intensification of the Detection Activities

In 2019, as part of the regular six-monthly meetings held by the entire Andalusian transplant coordination network, transplant coordinators from the main hospitals in Andalusia were asked to draw up a plan to increase tissue donation activity, and identify the hospital departments or units where tissue donor screening activities should be intensified, particularly regarding cornea donation, in accordance with the characteristics of each hospital and the staff who could collaborate in these activities.

### Data Collection and Analysis

For the purpose of this study we have included those deceased donors of somatic tissues except haematopoietic tissue, including donors of ocular tissues, skin, cardiovascular tissue and musculoskeletal tissue.

The tissue donation activity was analysed from 2019 to 2022. The information systems used included the Information System of the Regional Transplant Coordination of Andalusia (*Sistema de Información de la Coordinación Autonómica de Trasplantes de Andalucía, SICATA*), where all actual organ and/or tissue donors are registered, and the information management system of the Andalusian Tissue Banks (eProgesa) which registers data of utilised tissue donors and provides information on the effectiveness of the tissue donation process.

The evolution of tissue donation activity in general, and that of corneas in particular, was analysed by breaking down the type of donor, who may be a deceased organ donor who is brain dead or who died due to cardiocirculatory criteria who, in addition to organs, also donates tissues, or a donor exclusively of tissues.

**FIGURE 1:**
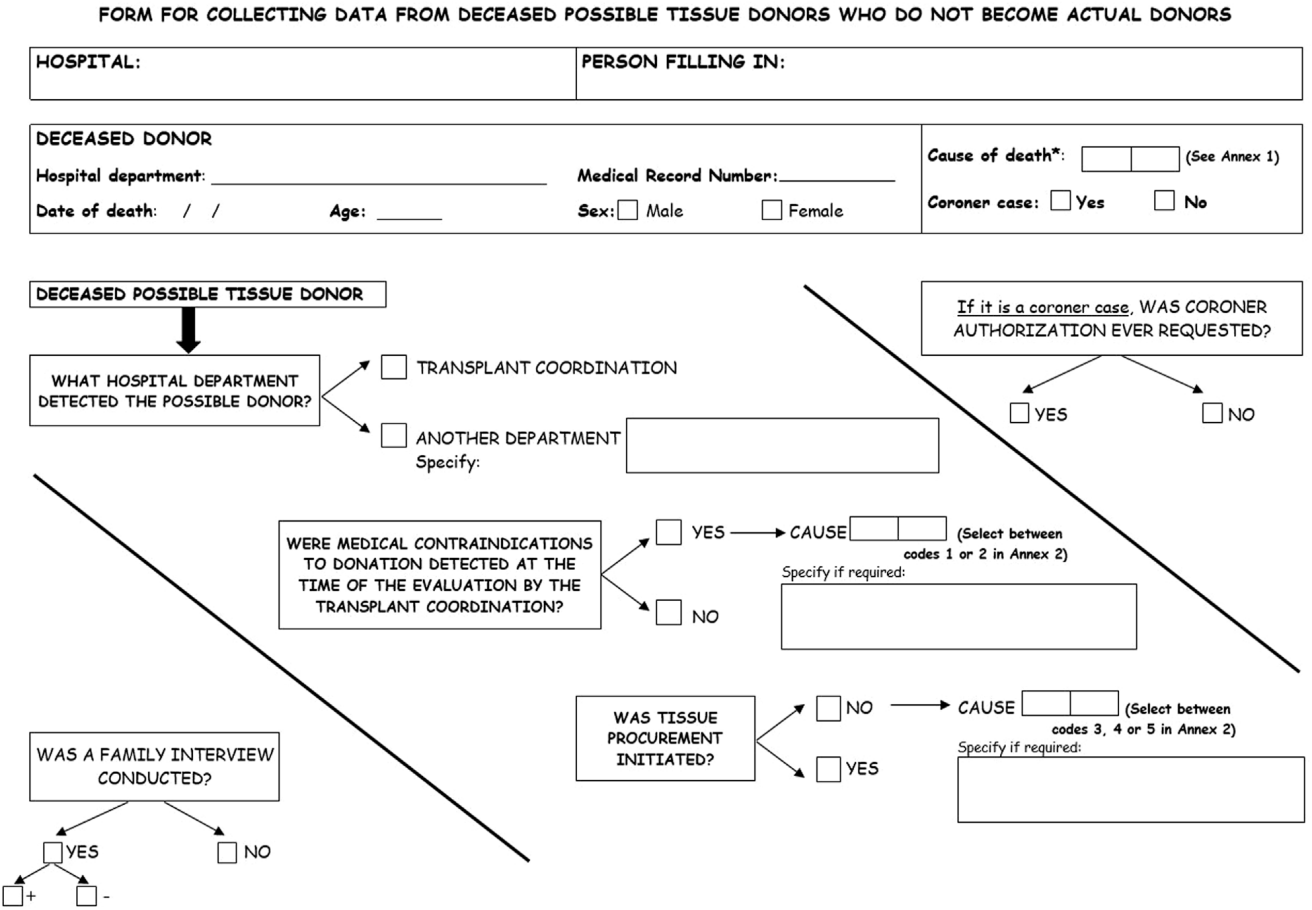
Front of the data collection form for deceased possible tissue donors who do not become actual donors.

**FIGURE 2:**
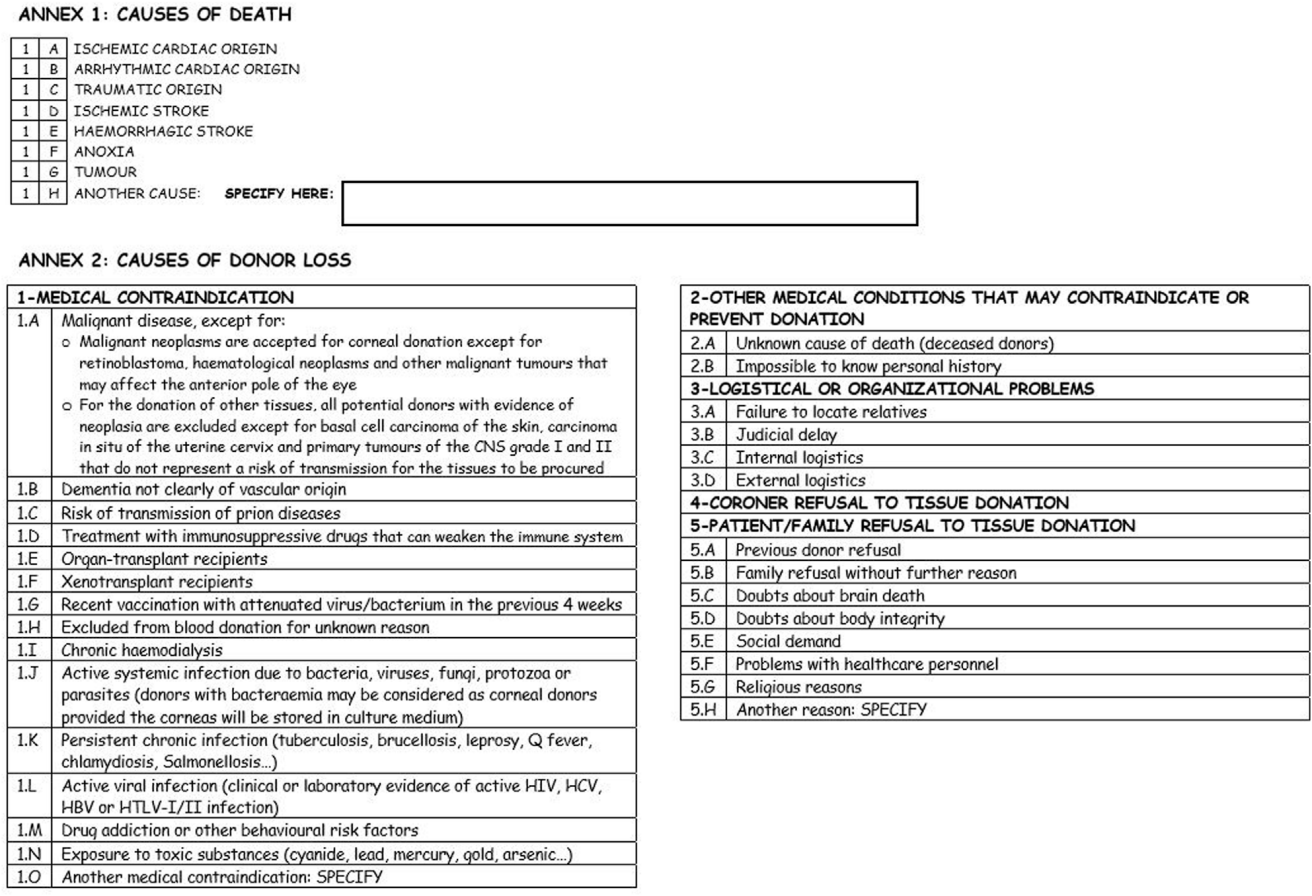
Back of the data collection form for deceased possible tissue donors who do not become actual donors.

In order to study the reasons for non-donation of tissue, the Regional Transplant Coordination (*Coordinación Autonómica de Trasplantes, CATA*) designed a form (Figures 1, 2) to collect data on deceased patients in the selected units who were identified as possible tissue donors, who ultimately did not donate. The form has been adapted from the one designed for the quality assurance programme for the organ donation process which was implemented in Andalusia in 1998. It includes information related to the hospital departments and healthcare professionals who identify possible donors, the possible donor’s characteristics, reasons for rejection, and information on family and legal interviews, if applicable. Data collection forms were always fulfilled by transplant coordinators reviewing medical charts and checking with the responsible doctor of the patient when necessary. An online form has also been developed to facilitate the collection and submission of data to the CATA.

Data collection on the reasons for non-donation of tissues started in 2020 and was analysed in 2022 using the critical pathway for deceased tissue donation methodology and the definitions listed in Table 1.

**TABLE 1:** Types of tissue donors. Definitions from the critical pathway for deceased tissue donation.

Types of tissue donors
**POSSIBLE TISSUE DONOR**: a person who has died (with death determined by neurological or circulatory criteria) or who is in a situation of imminent death
**POTENTIAL TISSUE DONOR**: a possible deceased donor with no apparent absolute contraindication for tissue donation and whose body has been preserved according to requirements for tissue procurement
**ELIGIBLE TISSUE DONOR**: a potential consented tissue donor who is medically suitable and meets specific criteria for the donation of at least one type of tissue
**ACTUAL TISSUE DONOR**: an eligible tissue donor from whom at least one tissue was recovered with the primary intention of clinical application
**UTILISED TISSUE DONOR**: an actual tissue donor from whom at least one, or part of a tissue is ready to be released for its clinical application

## Results

Since 2020, the implementation of local plans has been promoted and most hospitals have focused their detection activities on various intensive care units. In some hospitals, the accident and emergency department was involved, and in others, medical oncology wards were used to identify potential cornea donors. The units and departments were mainly selected based on whether the hospital ward supervisor nurse was able to collaborate.

**FIGURE 3:**
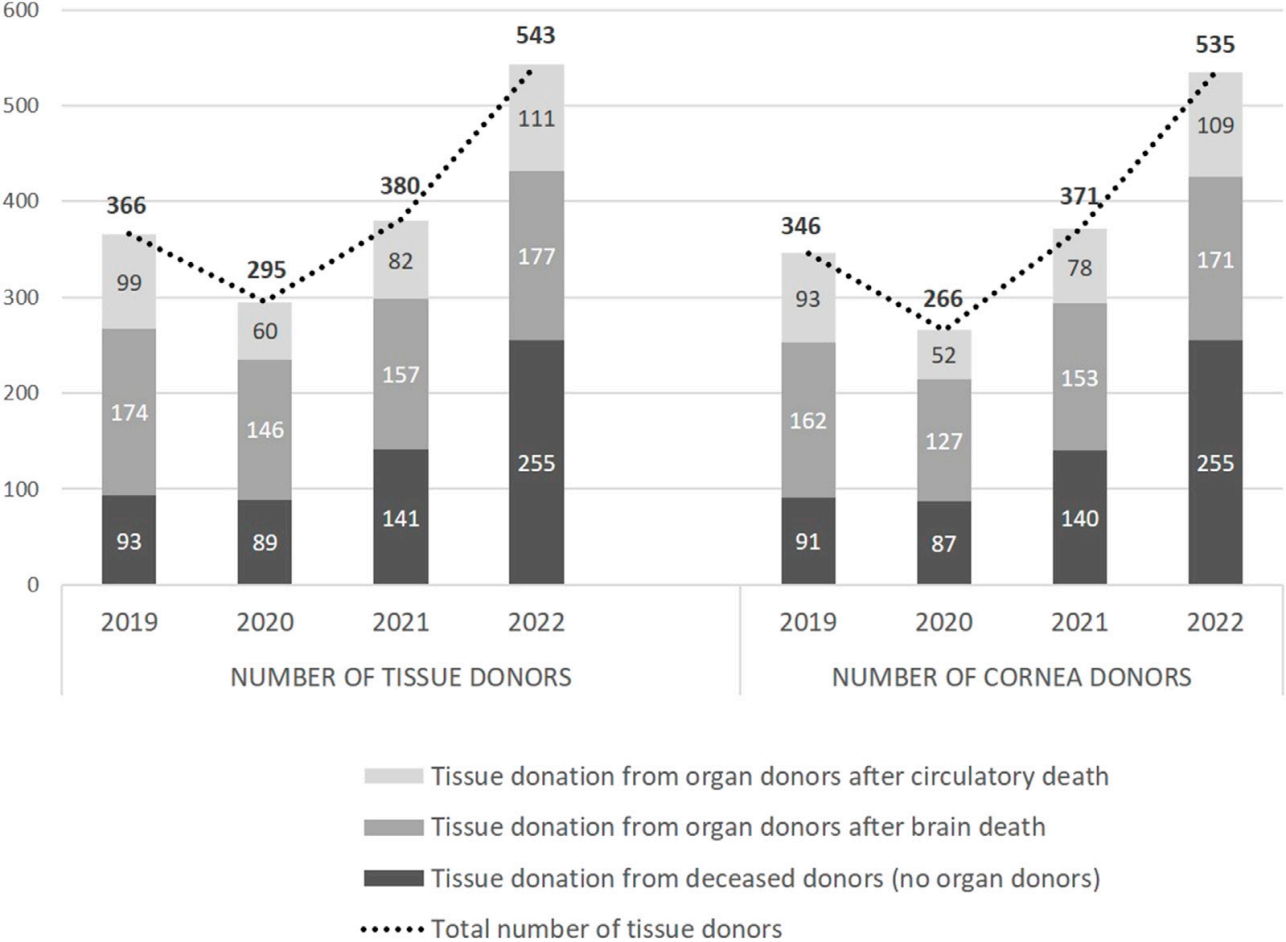
Change in the number of tissue donors and corneal donors from 2019 to 2022 in Andalusia according to the type of donor.

The change in the number of actual tissue donors and cornea donors from 2019, the year before the quality programme was implemented, to 2022, is shown in Figure 3. It also shows the total number of donors, and the number of donors in each of the following three categories: deceased donors who only donated tissues; brain-dead organ donors who also donated tissues; organ donors who also donated tissues after circulatory death.

Tissue donation in general, and cornea donation in particular, showed an important decline in 2020, which coincided with the outbreak of the coronavirus pandemic. However, in 2021, the activity exceeded that of 2019, and in 2022, once the pandemic was over, there was an extraordinary increase in activity compared to 2019. Specifically, the number of tissue donors increased from 366 to 543, which corresponds to an increase of 48.4%, and the number of cornea donors increased by 54.6% from 346 to 535 donors.

With regards to the type of donor, the number of tissue donors from brain-dead organ donors increased by 1.7%, 12.1% from organ donors after circulatory death, and 174% from deceased tissue-only donors. The increases were 5.6%, 17.2%, and 180.2% for the number of cornea donors in the same groups, respectively.

**FIGURE 4:**
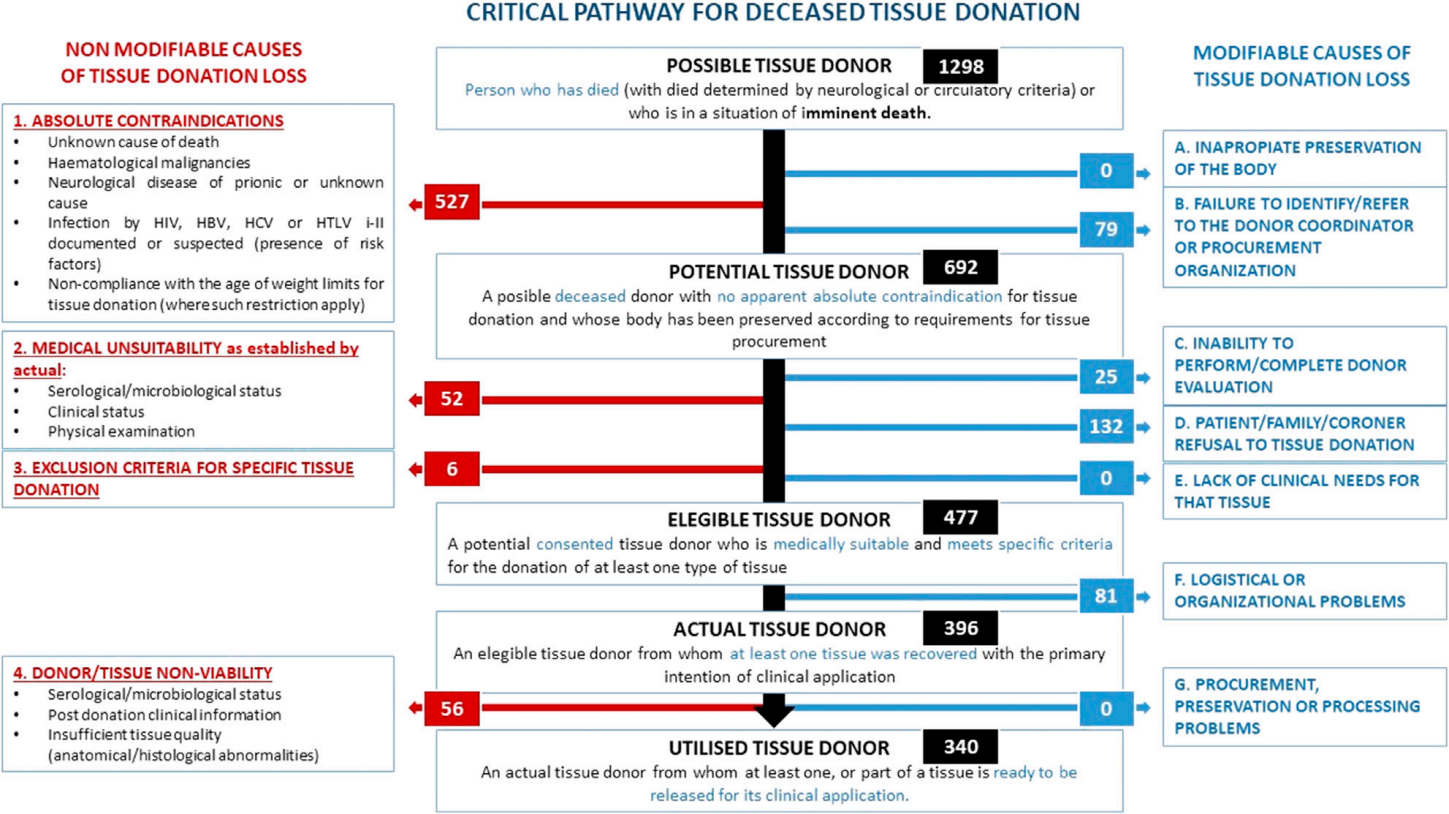
Causes of tissue donation loss according to the critical pathway for deceased tissue donation in Andalusia 2022.

Regarding the reasons for non-donation of tissues from possible donors identified in the selected units, although data collection started in 2020, uniform data collection did not begin until the end of the pandemic. Figure 4 shows the results obtained in 2022 and the reasons for non-donation according to the critical pathway definitions. This information comes from the registry of 902 possible tissue donors who ultimately did not donate, along with information from SICATA on 396 actual tissue donors in hospitals that participated in the data collection, for a total of 1,298 possible tissue donors. This information was collected in 18 of the 30 (60%) public hospitals in Andalusia that are authorised for organ donation and where 73% of the actual tissue donation activity occurs. The eProgesa data was then used to determine the number of utilized donors, 340.

Regarding the units and departments where 902 possible tissue donors were identified but ultimately did not donate, 71% were identified in different intensive care units, including coronary care and post-anaesthesia recovery units, 14% in the accident and emergency department, and 15% in hospital wards, not only in medical oncology but also in neurology, internal medicine, pneumology, haematology, neurosurgery, and orthopaedic surgery. The healthcare professionals who identified potential donors were transplant coordinators in 64% of cases, followed by intensive care doctors who were not part of the transplant coordination teams in 15% of cases, ward nurses in 6% of cases and the emergency department doctors in 4% of cases. The remaining 11% were identified by healthcare professionals from the aforementioned departments.

Of the 1,298 possible tissue donors (Figure 4), 527 had absolute contraindications to donation and 79 cases were not reported to the transplant coordinators of the hospital, bringing the number of potential tissue donors to 692. Of these, 477 were eligible donors because they were presumed medically suitable for the donation of at least one type of tissue, as well as having family consent for donation and, in judicial cases, also with judicial consent. The number of eligible donors who did not complete the donation process due to logistical or organisational issues was 81, bringing the number of actual donors, those from whom at least 1 tissue was procured, to 396, which corresponded to 83% of eligible donors. Finally, the number of utilised donors, those from whom at least part of a valid tissue was available to be released for clinical application, was 340, which corresponded to 85.9% of actual donors, and 71.3% of eligible donors.

Overall, around half of the possible donors (641, 49.4%) did not become utilised donors due to non-modifiable reasons. Of these, 527 were not donors because they had a previous absolute contraindication to tissue donation, to which 52 possible donors must be added, who were medically unsuitable, 6 who had some specific exclusion criterion for the tissue to be donated, and 56 who, after having some tissue recovered, were not viable for some of the reasons listed in Figure 4. Specifically, 26 were excluded due to known post-donation serological/microbiological status, 10 due to post-donation clinical information and 20 due to insufficient tissue quality.

The total number of possible donors who were not actual donors due to modifiable causes was 317, corresponding to about a quarter (24.4%). The modifiable causes, in order of frequency, were family refusal (132), followed by organisational or logistical issues (81), failure to notify or failure to identify possible donors (79) and failure to complete the donor evaluation (25).

## Discussion

The quality assurance programme for the tissue donation process was designed by the Regional Transplant Coordination of Andalusia and launched in 2020 thanks to the collaboration of the network of hospital transplant coordinators. This programme has been fundamental in increasing tissue donation activity in our region, so much so that 3 years after the start of its progressive implementation, activity has increased by almost 50% overall, and by more than 50% for cornea donation. This is despite the negative impact that the coronavirus pandemic had in 2020 and 2021 on donation and transplantation activity both in Spain and in most other countries [12, 13].

In fact, organ donation activity in Spain in 2020, 2021, and 2022, with 1,777, 1,905, and 2,196 organ donors respectively, represented 77.2%, 82.8%, and 95.4% of the activity recorded before the start of the pandemic in 2019 with 2,302 organ donors [2]. Similarly, in Andalusia, the number of organ donors changed from 430 in 2019 to 321, 335, and 415 donors in 2020, 2021, and 2022 respectively, representing 74.7%, 77.9%, and 96.7% of the activity in 2019.

The analysis of the change in tissue donation activity by type of donor shows that, in 2020 and 2021, there was a considerable decrease in the number of tissue donors who were also organ donors, with tissue-only donation maintaining similar figures in 2020 compared to 2019 and experiencing very substantial growth in 2021 that continued in 2022. It is possible that the initial recommendations of international [14] and national [15] organisations regarding the prioritisation of certain organ donation and transplantation programmes, and other substances of human origin, due to the risks of transmitting the infection, which were not well characterised in the early stages of the pandemic influenced the boost of tissue-only donation. The exhaustion of the health system and intensive care units during this initial period could be another factor.

The growth in tissue donation activity observed in 2022 cannot be attributed to the fact that the donation acceptance criteria were expanded between 2019 and 2022. Moreover, in 2022 the criteria were more restrictive than in 2019, given that COVID-positive tissue donors were still being rejected in that year. All other acceptance criteria remained unchanged. On the other hand, 2019 was the year that presented the best results in the history of Andalusia related to tissue donation, with an increase of 11% and 25% compared with the activity observed in 2018 and 2017 respectively, with no relevant growth in the population of Andalusia.

From the information collected through the specific form for potential tissue donors who ultimately did not donate, we have seen that identification is mainly performed in intensive care units by the transplant coordination teams, in many cases with the assistance of other intensive care specialists in these units. However, about 30% of potential unsuccessful donors were identified in the accident and emergency department and hospital wards as a result of the collaboration between several medical and nursing professionals. This data highlights the importance of training and close collaboration between transplant coordinators and other healthcare professionals, not only in intensive care units, but also in accident and emergency department and hospital wards, which is possibly the main specific area of growth for tissue donation activity.

The analysis of the results for the year 2022 according to the critical pathway for deceased tissue donation methodology developed by the CD-P-TO provides valuable information on the areas for improvement in tissue donation. Although the information comes from a sample of hospitals, where three-quarters of the donation activity took place, we believe that these preliminary data are noteworthy and, to our knowledge, are the first to be published internationally using this methodology. They may therefore become a benchmark to facilitate comparative analysis with other regions or countries as they implement the critical pathway for deceased tissue donation methodology.

We have found that tissue donation activity could be substantially increased if we could reverse the modifiable causes of donor loss, although reversing some of these causes is partly dependent on factors beyond the control of the transplant coordination network, or difficult to control such as family refusal, which is the main modifiable cause of loss of possible tissue donors. However, there are other modifiable causes that are easier to address. Losses due to organisational and logistical reasons, which in some cases are due to limitations in the availability of operating theatres or human resources, require more detailed analysis and support from hospital managers in order to be reversed, as in the case of failure to complete donor evaluation. Meanwhile, the lack of notification or failure to identify the possible donors could be more easily rectified by establishing a closer relationship with the units that could potentially provide tissue donors, improving the training of the professionals working in these units and implementing fast notification systems. In 2022, the Regional Transplant Coordination of Andalusia, which promotes and develops important training activity [16], launched a large-scale virtual training programme on general aspects of donation and transplantation, which in its first year was taken by more than 1,800 professionals from the Andalusian Health Service. This large-scale training programme has been added to the 19 training courses already in place on specific aspects of donation and transplantation, and we hope it will lead to an increase in the identification of possible donors.

Regarding the efficacy of the tissue donation process, 396 of the 1,298 possible donors were actual donors, which represents 30.5%. If we compare these results with the most recent results of the quality assurance programme for the organ donation process in Spain for 2021 [17], we see that this efficacy is much lower than that observed in organ donation, where 48.8% of possible donors become actual donors. However, it should be noted that the medical criteria for tissue donation are more stringent [18] than those for organ donation [19], and therefore part of this difference in efficacy is due to a higher percentage of rejected cases due to lack of medical suitability for donation. This resulted in 34.6% of possible organ donors being rejected due to lack of medical suitability in Spain in 2021, while the percentage of possible tissue donors rejected for medical reasons (527 possible donors with absolute contraindications, 52 medically unsuitable and 6 with exclusion criteria for some type of tissue) amounted to 45.1% in our sample. Related to the other causes of loss of possible donors, when comparing organ and tissue donation, we found that losses due to family or legal refusal were 10.2% for tissue donation compared to 10.6% for organs, losses due to logistical or organisational issues were 6.2% compared to 0.3%, and losses due to identification notification failures were 6.1% compared to 0.8%, respectively.

The analysis of effectiveness, i.e., the percentage of actual tissue donors who became utilised donors, amounted to 86% in 2022 in our analysed sample. This figure was slightly higher than the 85% effectiveness achieved in Andalusia in organ donation and was slightly lower than the 89% effectiveness achieved in Spain [2] in the same year.

A notable aspect is the optimisation of the resources involved in the implementation of the programme, as it has been carried out without increasing the number of staff. This has been made possible thanks to the efforts of the network of transplant coordinators and the collaboration of many healthcare professionals who are not involved in the donation and transplant programmes. In some cases, these collaborators performed their care activities in units other than those traditionally involved in organ procurement, such as medical oncology departments in the case of corneal donation.

Finally, it is important to emphasise that our tissue donation quality assurance programme, supported by the information systems in Andalusia, has allowed us not only to increase tissue donation activity, but also to evaluate the potential and effectiveness of the tissue donation process. It is also useful to establish benchmarks for comparison between donation centres as well as for identifying areas and measures for improvement, which we hope will lead to continuous improvement of the process.

## Data Availability

The original contributions presented in the study are included in the article, further inquiries can be directed to the corresponding authors.

## Abbreviations

CATA, Coordinación Autonómica de Trasplantes de Andalucia; TC, Transplant Coordinator; CD-P-TO, European Committee on Organ Transplantation of the Council of Europe; eProgesa, Information management system of the Andalusian Tissue Banks; SICATA, Sistema de Información de la Coordinación Autonómica de Trasplantes de Andalucía.
